# The complete chloroplast genome sequence of corn and economic analysis on production costs profits in Zhengzhou city

**DOI:** 10.1080/23802359.2020.1775524

**Published:** 2020-06-16

**Authors:** Yiwei Chen, Yongshuang Ma, Xueli Xu

**Affiliations:** a School of Economics and Management, Southeast university, Nanjing, China; b Institute of Rule of Law for Rural Revitalization, Zhejiang Agricultural and Forestry University, Hangzhou, China; c School of Biological and Chemical Engineering, Nanyang Institute of Technology, Nanyang, China

**Keywords:** Corn, chloroplast genome, phylogenetic analysis, genetic information

## Abstract

Corn is one of the main food crops in China. The problem of corn production cost benefit has always been the most concerned issue of farmers. In this paper, economic analysis of corn production cost benefit is conducted, and the chloroplast genome sequence is studied. The complete chloroplast genome sequence of corn was characterized from Illumina pair-end sequencing. The chloroplast genome of corn was 140,382 bp in length, containing a large single-copy region (LSC) of 82,156 bp, a small single-copy region (SSC) of 15,952 bp, and two inverted repeat (IR) regions of 21,137 bp. The overall GC content is 36.80%, while the correponding values of the LSC, SSC, and IR regions are 34.5%, 30.5%, and 42.5%, respectively. The genome contains 118 complete genes, including 85 protein-coding genes (78 protein-coding gene species), 25 tRNA genes (19 tRNA species) and 8 rRNA genes (4 rRNA species). The Neighbour-joining phylogenetic analysis showed that corn and *Zea mays* cultivar B73 clustered together as sisters to other *Zea* species.

## Introduction

Corn was one of the most important and ancient domesticated crops in the world. Henan province is one of the largest corn producing provinces in China. The annual planting area of corn in the province is about 2.853 million hm^2^, accounting for about 25% of the planting area of crops. In particular, Zhengzhou’s corn output accounts for 13% of the total corn output of Henan province. It is not only self-sufficient, but also can be continuously transferred abroad. Therefore, Zhengzhou’s corn market development occupies a very important position in the whole province and even the national corn market. But the cost of growing corn remains high and does not bring farmers much profit. Through the analysis of the planting cost structure of corn, the reasons for the high production cost of corn are obtained, and the cost and benefit of other crops are compared. Moreover, we can develop conservation strategies easily when we understand the genetic information of corn. In the present research, we constructed the whole chloroplast genome of corn and understood many genome varition information about the species, which will provide beneficial help for population genetics studies of corn.

The fresh leaves of corn were collected from Zhengzhou (112°42′E; 34°16′N). Fresh leaves were silica-dried and taken to the laboratory until DNA extraction. The voucher specimen (YM001) was laid in the Herbarium of Nanyang Institute of Technology and the extracted DNA was stored in the −80 °C refrigerator of the Key Laboratory of School of Biological and Chemical Engineering. We extracted total genomic DNA from 25 mg silica-gel-dried leaf using a modified CTAB method (Doyle 1987). The whole-genome sequencing was then conducted by Biodata Biotechnologies Inc. (Hefei, China) with Illumina Hiseq platform. The Illumina HiSeq 2000 platform (Illumina, San Diego, CA) was used to perform the genome sequence. We used the software MITObim 1.8 (Hahn et al. 2013) and metaSPAdes (Nurk et al. 2017) to assemble chloroplast genomes. We used *Zea mays* cultivar B73 (GenBank: KF241981) as a reference genome. We annotated the chloroplast genome with the software DOGMA (Wyman et al. 2004), and then corrected the results using Geneious 8.0.2 (Campos et al. 2016) and Sequin 15.50 (http://www.ncbi.nlm.nih.gov/Sequin/).

The complete chloroplast genome of corn (GenBank accession number MT408562) was characterized from Illumina pair-end sequencing. The complete chloroplast genome sequence of corn was characterized from Illumina pair-end sequencing. The chloroplast genome of corn was 140,382 bp in length, containing a large single-copy region (LSC) of 82,156 bp, a small single-copy region (SSC) of 15,952 bp, and two inverted repeat (IR) regions of 21,137 bp. The overall GC content is 36.80%, while the correponding values of the LSC, SSC, and IR regions are 34.5%, 30.5%, and 42.5%, respectively. The genome contains 118 complete genes, including 85 protein-coding genes (78 protein-coding gene species), 25 tRNA genes (19 tRNA species) and 8 rRNA genes (4 rRNA species).

**Figure 1.**
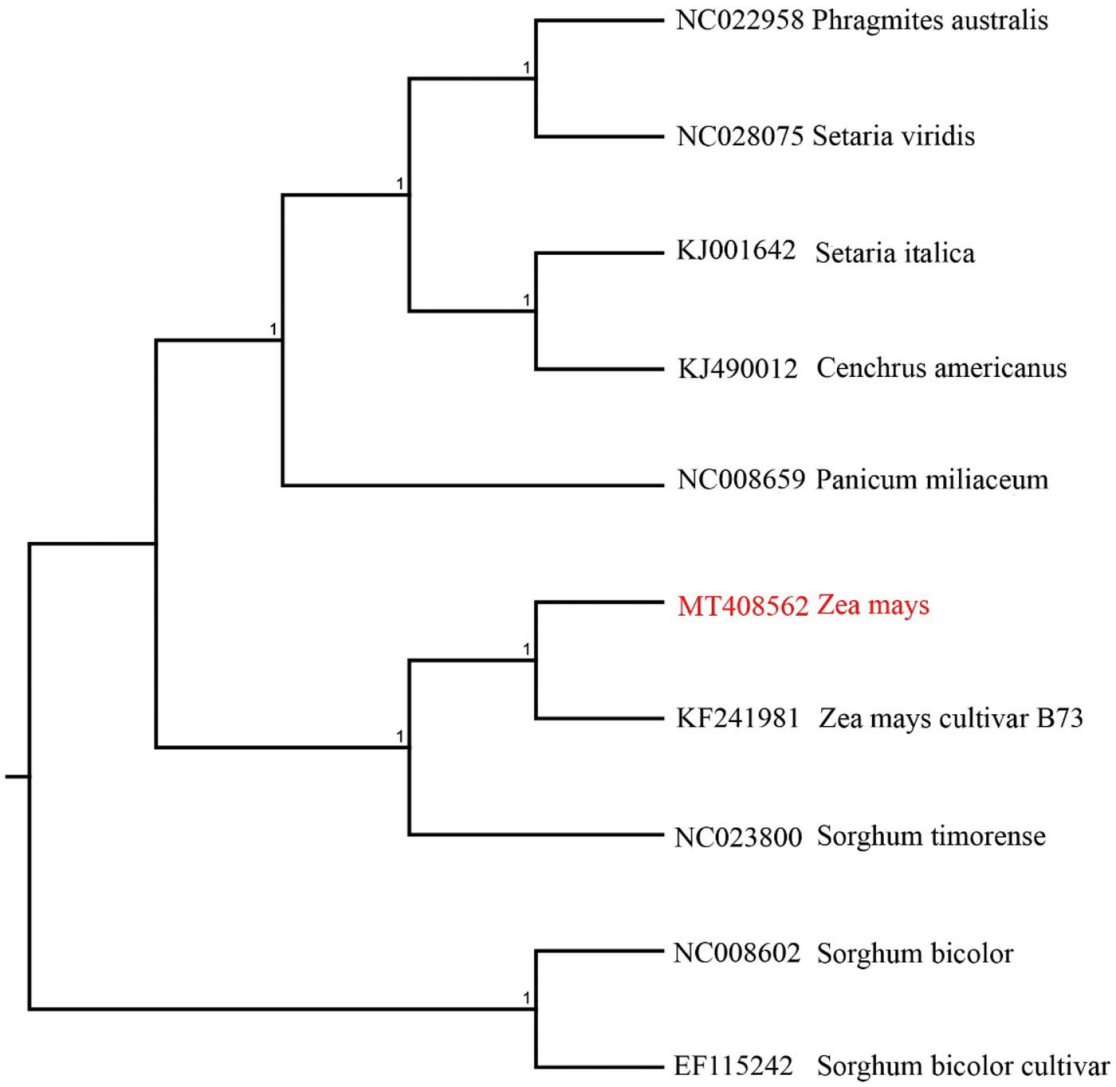
Neighbour-joining (NJ) analysis of corn and other related species based on the complete chloroplast genome sequence. *Sorghum bicolor* (NC008602) was set as the outgroup. All other sequences were downloaded from NCBI GenBank.

We used the complete chloroplast genomes sequence of corn and 9 other related species of *Zea* and *Sorghum bicolor* as outgroup to construct phylogenetic tree. The 10 chloroplast genome sequences were aligned with MAFFT (Katoh and Standley 2013), and then the Neighbour-joining tree was constructed by MEGA 7.0 (Kumar et al. 2016). The results confirmed that corn and *Zea mays* cultivar B73 clustered together as sisters to other *Zea* species (Figure 1).

## Data Availability

The data that support the findings of this study are openly available in National Center for Biotechnology Information (NCBI) at https://www.ncbi.nlm.nih.gov, accession number MT408562.
